# Stereotactic ablative brachytherapy versus percutaneous microwave ablation for early-stage non-small cell lung cancer: a multicenter retrospective study

**DOI:** 10.1186/s12885-024-12055-6

**Published:** 2024-03-06

**Authors:** Chuanwang Wu, Binglong Cao, Guanghui He, Yuliang Li, Wujie Wang

**Affiliations:** 1 Department of Interventional Medicine,The Second Hospital of Shandong University, Institute of Tumor Intervention,Cheeloo college of medicine, Shandong University, Jinan City, Shandong Province China; 2 Department of Fifth Internal Medicine, People’s Hospital of Shizhong District, No.156 Jiefang Road, Zaozhuang City, Shandong Province China; 3 Department of Oncology, Qufu Hospital of Traditional Chinese Medicine, No.129 Canggeng Road, Qufu City, Shandong Province China; 4 Department of Interventional Medicine, Weifang Second People’s Hospital, Weifang city, Shandong Province China

**Keywords:** Stereotactic ablative brachytherapy, Microwave ablation, Percutaneous, Early stage, Non-small cell lung cancer, Comparative study

## Abstract

**Background:**

To analyze the efficacy of stereotactic ablative brachytherapy (SABT) and percutaneous microwave ablation (MWA) for the treatment of early-stage non-small cell lung cancer (NSCLC).

**Methods:**

Patients with early-stage (T1-T2aN0M0) NSCLC who underwent CT-guided SABT or MWA between October 2014 and March 2017 at four medical centers were retrospectively analyzed. Survival, treatment response, and procedure-related complications were assessed.

**Results:**

A total of 83 patients were included in this study. The median follow-up time was 55.2 months (range 7.2–76.8 months). The 1-, 3-, and 5-year overall survival (OS) rates were 96.4%, 82.3%, and 68.4% for the SABT group (*n* = 28), and 96.4%, 79.7%, and 63.2% for MWA group (*n* = 55), respectively. The 1-, 3-, and 5-year disease-free survival (DFS) rates were 92.9%, 74.6%, and 54.1% for SABT, and 92.7%, 70.5%, and 50.5% for MWA, respectively. There were no significant differences between SABT and MWA in terms of OS (*p* = 0.631) or DFS (*p* = 0.836). The recurrence rate was also similar between the two groups (*p* = 0.809). No procedure-related deaths occurred. Pneumothorax was the most common adverse event in the two groups, with no significant difference. No radiation pneumonia was found in the SABT group.

**Conclusions:**

SABT provided similar efficacy to MWA for the treatment of stage I NSCLC. SABT may be a treatment option for unresectable early-stage NSCLC. However, future prospective randomized studies are required to verify these results.

## Introduction

Lung cancer remains the leading cause of cancer-related death worldwide and will result in more than 350 deaths each day in 2022 in the United States [1]. Non-small cell lung cancer (NSCLC) accounts for approximately 85% of all cases [2]. With the increasing implementation of screening protocols using low-dose computed tomography, rising rates of early-stage lung cancer have been detected [3]. Surgical resection remains the cornerstone for operable early-stage NSCLC [4]. However, severe medical comorbidities, primarily poor cardiopulmonary function, prevent more than 25% of early-stage NSCLC patients from undergoing surgery despite the advancement of sub-lobular resection (SLR) to reduce the effects of surgery on lung function [4, 5].

Current treatment options for early-stage NSCLC ineligible to undergo surgical resection mainly include stereotactic ablative radiotherapy (SABR) and thermal ablation (TA) [6–9]. Various ablation modalities have been used to deliver heat- or cold-induced cancer cell death [10]. Microwave ablation (MWA) has emerged as an alternative option for early-stage NSCLC in recent years and is associated with similar clinical outcomes to lobectomy [11–14]. More recently, stereotactic ablative brachytherapy (SABT) was introduced to expand the treatment choices for early-stage NSCLC [15]. Stereotactic ablative brachytherapy (SABT) had primarily been accomplished through the direct implantation of radioactive seeds into malignant tissues. This procedure circumvented the dose fluctuations associated with tumor movement during spontaneous respiration. Typically, high radiation doses ranging from approximately 110 to 160 Gy were necessitated for effective SABT, potentially resulting in complete tumor ablation. The success of SABT crucially depended on organ preservation and was contingent upon several pivotal technological components, notably precise image guidance and accurate dose calculations [16, 17]. SABT has evolved as a safe and effective method for the treatment of various malignant tumors in recent years [18–20], including early-stage NSCLC [21]. Moreover, performing SABT only once for early-stage NSCLC is sufficient, which requires relatively simple facilities without an expensive linear accelerator; this, in turn, facilitates the patient’s medical treatment and allows the procedure to be carried out in primary hospitals. For these reasons, several patients of inoperable early-stage lung cancer receive SABT in real-world clinical practice.

To the best of our knowledge, no comparative study about the effectiveness of MWA and SABT for early-stage NSCLC has been reported. In this retrospective study, we aimed to analyze the effectiveness of SABT for stage T1-T2bN0M0 NSCLC in comparison to MWA in four medical centers.

## Materials and methods

### Patients

Patient data for this retrospective analysis were obtained from the database of four medical centers. We identified all patients that received MWA or SABT for stage T1–2aN0M0 NSCLC from October 2014 to November 2017. Case selection criteria were: (1) NSCLC diagnosed pathologically by biopsy or cytology at the first visit; (2) Classified as stage T1–2aN0M0 (stage Ia–Ib) NSCLC after systemic evaluation with computed tomography (CT) and/or positron emission tomography CT (PET-CT) based on the American Joint Committee on Cancer (AJCC) TNM Classification 8th edition [22]; (3) All cases were evaluated at a multidisciplinary team (MDT) meeting including a thoracic surgeon and deemed unsuitable for surgery because of baseline forced expiratory volume in one second (FEV1) < 1.5 L, pre-operative FEV1 < 40% predicted value or diffusing capacity of the lung for carbon monoxide (DLCO) < 40% of predicted value, diabetes mellitus with organ damage, severe cerebral or cardiovascular disease, or severe pulmonary hypertension; (4) MWA or SABT was the initial treatment.

The treatment was administered after sufficiently informing the patients and their immediate relatives about the risks and benefits of SABT and MWA. Written informed consent was obtained from all patients. The study followed the ethical guidelines of the 1975 Declaration of Helsinki and also was approved by the Ethical Committees of the hospitals.

### SABT

#### Preoperative preparation of SABT

Contrast-enhanced CT (CECT) was performed 1 week before the procedure. After the transmission of the image data to the treatment planning system (TPS) (Beijing University of Aeronautics and Astronautics and Beijing Astro Technology Ltd, Co., Beijing, China; Version 7.4.), delineation of the clinical target volume (CTV) was performed by the specialized medical physicist and radiologist together using slices with a 5-mm thickness. CTV was defined as 3 mm of expansion to the gross target volume (GTV), which was contoured. The prescription dose was planned according to the CTV and empirically set as 110–160 Gy. The specific dose was determined based on the size of the tumor and the location with the adjacent organ at risk. The seed radioactivity was 0.5–0.6 mci with an average energy of 27–35 keV. The seed number, puncture direction, and insertion depth were planned.

The iodine-125 seeds (half-life, 59.6 days; in cylinder shape; 0.8 mm in diameter; length, 4.5 mm; Jaco Pharmaceuticals Co., Ltd., Ningbo, China) were measured with a radioactivity meter (Yida Measurement Technology Co., Ltd., Beijing, China) before the procedure to ensure the variation between calculated and measured activities was less than 5%.

#### Brachytherapy procedure

**Fig. 1:**
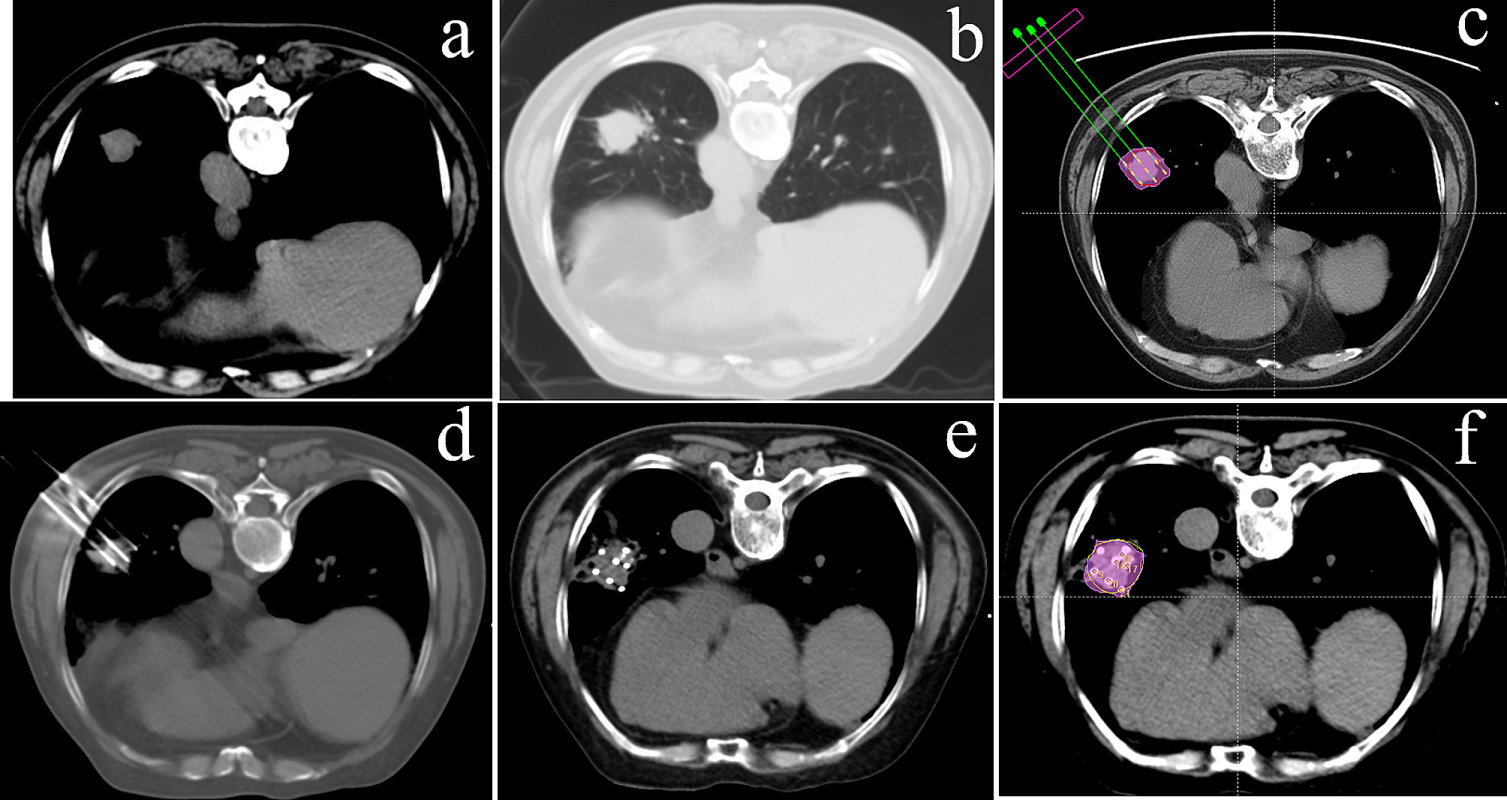
Flow chart of SABT: (**a**) target lesion (mediastinal window); (**b**) target lesion (lung window); (**c**) preoperative plan; (**d**) intraoperative needle insertion under CT guidance; (**e**) seeds implantation; (**f**) dose verification

All the procedures were performed by interventional radiologists who had more than 15 years of experience in seed implantation under intravenous anesthesia. A vacuum cushion was used to fix the patients in the planned position. Then, disposable 18-gauge coaxial needles (Hakko Co., Ltd.) were inserted into the target lesion under CT guidance with the needle tips positioned at the distal edge of the tumor according to the preoperative plan. Then, the planned number of iodine-125 seeds was implanted into the target area. After seed implantation, a CT scan was performed to verify the spatial distribution of seeds and dose distribution in the target lesion. Additional seeds were implanted if a cold area(s) existed. A flow chart relating to SABT is shown in Fig. 1.

#### Postoperative management

The patients were placed in special areas with radiation warnings after the procedure. Radiation protection pads (containing 0.25 mm lead) were worn by the patients. A chest radiograph was performed 24 h after the procedure to check for pneumothorax.

### Microwave ablation

The MWA procedure was performed under CT (Lightspeed 64 V, GE General Electric) guidance with intravenous conscious sedation using dexmedetomidine. The vital signs of the patients were continuously monitored by a dedicated anesthetist during the procedure. All the MWA ablations were performed by interventional radiologists who had more than 15 years of experience in thermal ablation. An ECO-100A1 microwave therapeutic system (ECO, Microwave Electronic Institute, Nanjing, China) with 2450 ± 20 MHz was used in this study. The effective length and outside diameter of the microwave antenna were 100–180 mm and 16–19 G, respectively. When the output was 40 W/10 min, the ablative zone was approximately 4.5 cm × 3.5 cm. A CT scan was performed after the anesthesia team was satisfied. The puncture point, direction, and depth were planned. Then, the microwave antenna was inserted into the lesion under CT guidance. The planned tip of the antenna was placed at the distal edge of the lesion. Before ablation, the cooling equipment, including circulating pipes and pumps, was connected to the antennae for circulating cooling to the surface temperature of the antennae. The output was set according to the location and size of the lesions. The area of exudative change around the target lesion with 5–10 mm expansion was considered a technical success. One antenna was used for lesions no larger than 3 cm. If the tumor was larger than 3 cm, two parallel antennae were inserted into the target lesion to achieve technical success. After ablation, the puncture track was routinely coagulated. A chest radiograph was performed the next day to check for pneumothorax.

### Endpoints

The primary endpoint of this study was OS. Secondary endpoints were disease-free survival (DFS) and procedure-related adverse events. OS was defined as the date of the procedure to the date of death or last follow-up. DFS was defined as the date of the procedure to local recurrence or distant recurrence.

For SABT, treatment response was evaluated according to the International Response Evaluation Criteria in Solid Tumor (RECIST) [23]. Complete ablation was confirmed if there was no contrast enhancement displayed in any of the ablation areas. Local recurrence was confirmed if follow-up images showed new focal enhancement or increased fluorodeoxyglucose (FDG) uptake in the ablation area or the margins. Distant recurrence was confirmed if new lesions were detected in another site besides the primary lesion [24, 25]. Regional nodal failure was confirmed if recurrence occurred within a different ipsilateral lobe or any regional lymph node station, according to the AJCC Cancer Staging Manual, 7th edition. Procedure-related adverse reactions were classified as mild events, moderate events, severe events, disabling events, and patient death based on criteria from the Society of Interventional Radiology (SIR) [26].

### Follow-up

For the first year after the procedure, CECT was performed every month for the first 3 months, then every 3 months for the remainder of the year. After that, follow-up CECT was performed every six months. PET-CT or biopsy was performed if a potentially recurrent or residual tumor was detected. All conclusions about the follow-up imaging were made with the consensus of two radiologists with more than 15 years of experience.

### Statistical analysis

Statistical analyses were performed using SPSS 24.0 software (IBM Corp., Armonk, NY, USA). For quantitative data (tumor size, age), independent t-tests were used to identify differences between the SABT and MWA groups; For comparison of qualitative data (gender, tumor location, histologic type, tumor stage), a chi-square test was used; For survival analysis (OS, DFS), the Kaplan–Meier method with log-rank test was used. To analyze the factors that impact primary and secondary outcomes, a mixed linear model was utilized. The fixed effects were histologic type, age, smoking history, gender, and tumor size. The treatment sites were considered as random effects. Chi-square tests were also used to compare the complication and recurrence rates of the two groups. A p value of < 0.05 was deemed statistically significant.

## Results

### Patient and tumor characteristics

A total of 83 patients were included in this study after screening. There were 55 patients that received MWA and 28 that received SABT. Only 12 patients (14.5%) were staged by PET-CT because of the expensive nature of the test and lack of medical insurance payments. The median age of this group was 63 years (range, 35–77 years), and the median tumor size was 2.9 cm (range, 1.3–4.0 cm). Histological subtypes were 44 cases (53.0%) of adenocarcinoma, 33 cases (39.8%) of squamous cell carcinoma and 6 cases (7.2%) of adenosquamous carcinoma. No significant difference was identified in any of the baseline characteristics between the SABT and MWA groups (Table 1).

**Table 1:** Patients baseline data

Characteristics	SABR Group (*n* = 28)	MWA Group (*n* = 55)	*P*
Age (Mean ± SD)	63.82 ± 9.23	59.75 ± 10.47	0.074
Gender			0.824
Male Female	21 7	40 15	
Max. Tumor Size (cm, Mean ± SD)	2.96 ± 0.55	2.89 ± 0.66	0.567
Tumor Staging			0.818
Ia Ib	15 13	28 27	
Histologic type			0.856
AD	16	28	
SQ	10	23	
AS	2	4	
Tumor location^a^			0.694
Central	7	16	
Peripheral	21	39	

Independent t-test was used for quantitative data (tumor size, age); Chi-square test was used for qualitative data (gender, tumor location, histologic type, tumor stage); AD, adenocarcinoma; SQ, squamous cell carcinoma; AS, adenosquamous carcinoma. ^a^ Central was tumor located at or above pulmonary segment on CT. Peripheral was tumor located below pulmonary segment on CT.

### Seed implantation

A median number of 36 (range, 22–50) iodine-125 seeds (median radioactivity, 0.7 mCi; range, 0.6–0.8 mCi) were implanted in the SABT group. Dose verification after the procedure showed the median D90 (dose absorbed by 90% of GTV) and Dmax were 155.62 Gy (range, 143.66–180.79 Gy) and 1464.32 Gy (range, 1370.25–1796.18 Gy), respectively. Median conformity index (CI), external index (EI), and homogeneity index (HI) were 0.64 (range, 0.56–0.81), 0.30 (range, 0.14–0.54), and 0.35 (range, 0.26–0.43), respectively.

### Treatment response

**Fig. 2:**
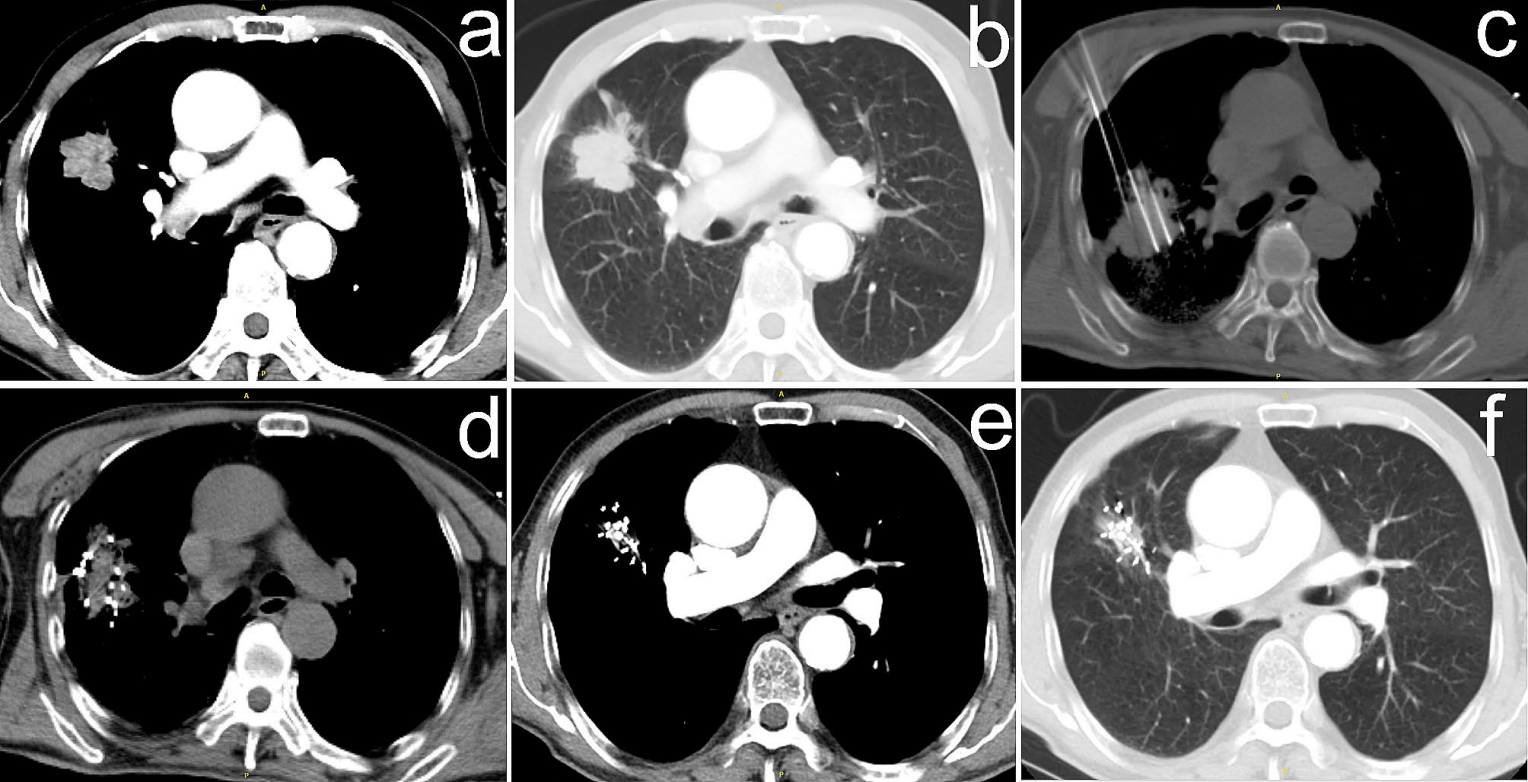
(**a**) Thoracic CT demonstrated a mass in the right lung inferior lobe before treatment; (**b**) Lung window; (**c**) Multiple needles were inserted into the lesion under CT guidance; (**d**) Iodine-125 seeds were implanted into the tumor; (**e**) Follow-up CT 24 months after the procedure showed complete response; (**f**) Lung window

**Fig. 3:**
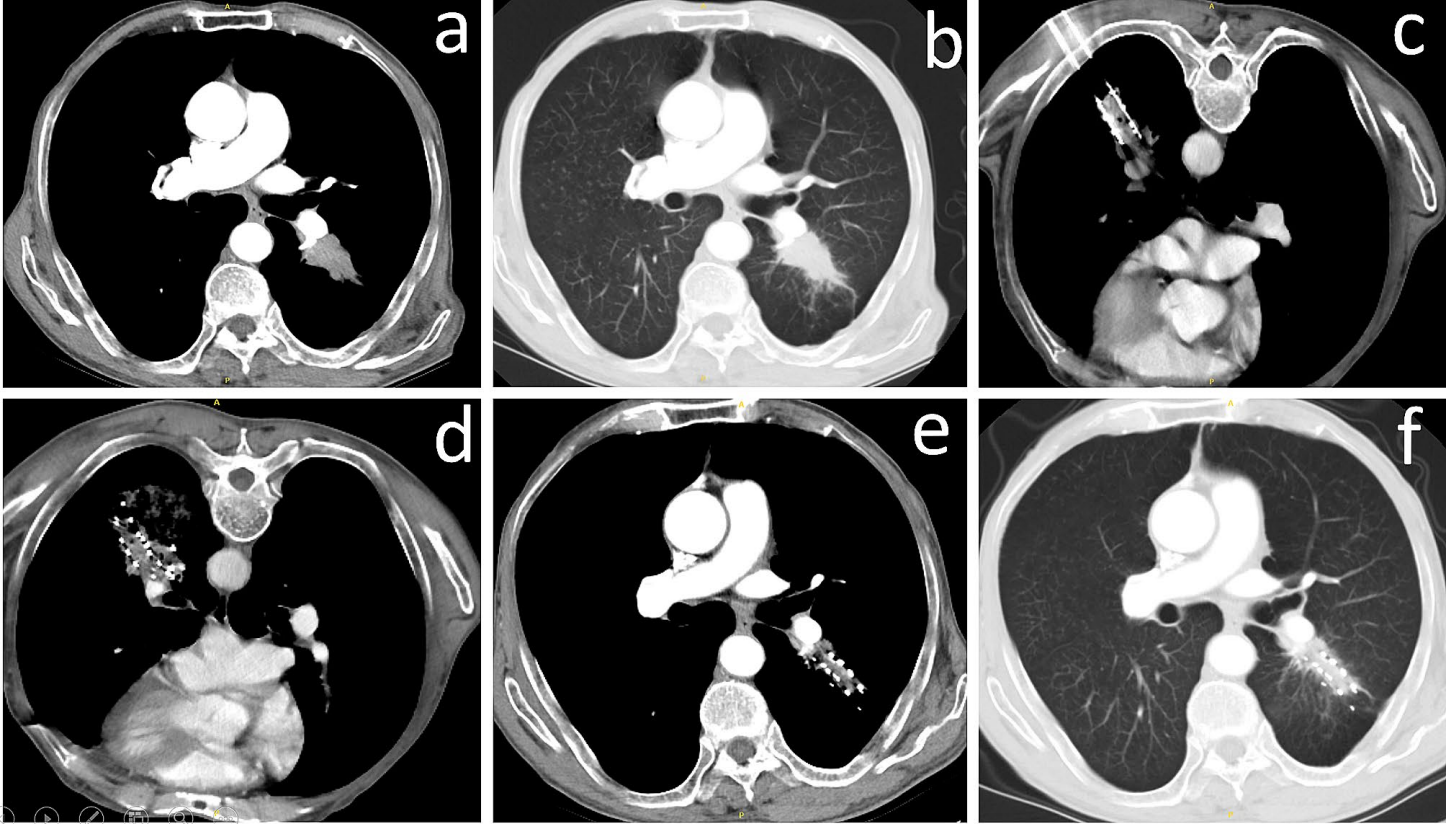
(**a**) Thoracic CT showed a lesion adjacent to pulmonary artery in the left lung; (**b**) Lung window;(**c**) Multiple needles were inserted into the lesion under CT guidance;(**d**) Iodine-125 seeds were implanted into the tumor;(**e**) Local control was observed during a Follow-up of 34 months;(**f**) Lung window

Of all 83 patients, local recurrence occurred in 19 (22.9%), while local control was observed in 64 (77.1%) over a median follow-up duration of 55.2 months (range, 7.2–76.8 months) (Figs. 2 and 3).

In the SABT group (*n* = 28), 15 (53.6%) patients experienced disease progression during follow-up, including local recurrence in 4 cases (14.3%), distant recurrence in 9 cases (32.1%), and both local and distant recurrence in 2 cases (7.2%). Regional nodal failure was detected in 5 (17.9%) of the 11 cases with distant recurrence.

In the MWA group (*n* = 55), 31 (56.4%) patients experienced tumor recurrence, including 8 (14.6%) patients with local recurrence, 18 (32.7%) patients with distant recurrence, 5 (9.1%) patients with both local and distant recurrence. Regional nodal failure was detected in 12 (21.8%) of the 23 cases with distant recurrence.

The was no significant difference in recurrence rates between the two groups (*p* = 0.809).

Five patients in the SABT group and 12 patients in the MWA group who experienced distant recurrence received chemotherapy with docetaxel or gemcitabine and/or platinum-based drugs. Target therapy was used in 3 patients in the SABT group and 6 patients in the MWA group.

### Survival

The median OS and DFS were 5.70 ± 0.28 years (95% CI: 5.15, 6.25 years) and 5.10 ± 0.27 years (95% CI: 4.58, 5.63 years), respectively, in the SABT group and 5.80 ± 0.34 years (95% CI: 5.14, 6.47 years) and 5.20 ± 0.46 years (95% CI: 4.30, 6.10 years), respectively, in the MWA group.

**Fig. 4:**
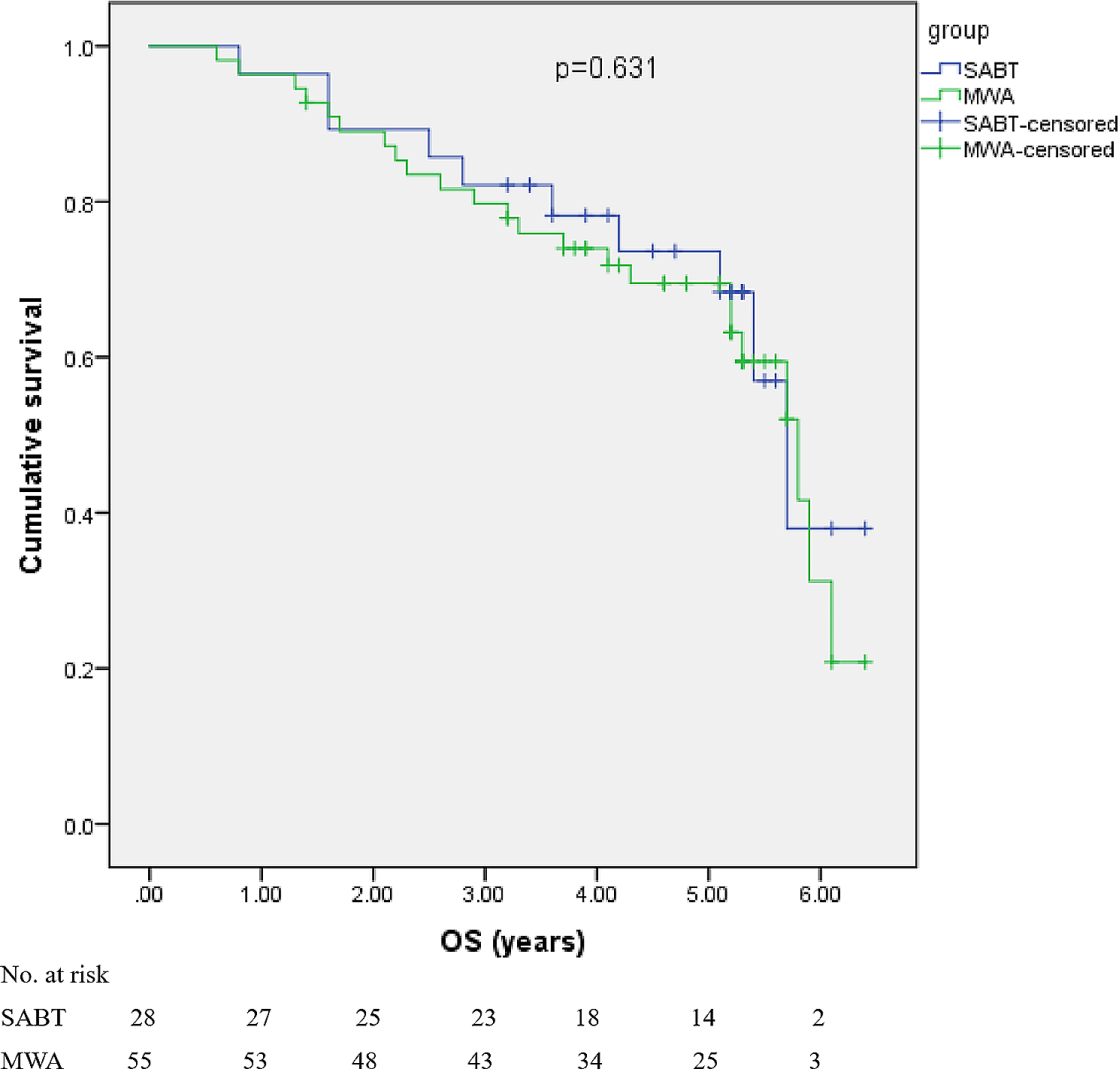
Kaplan-Meier curves of overall survival comparison between the SABT and MWA groups. There was no significant difference (*p* = 0.631)

**Fig. 5:**
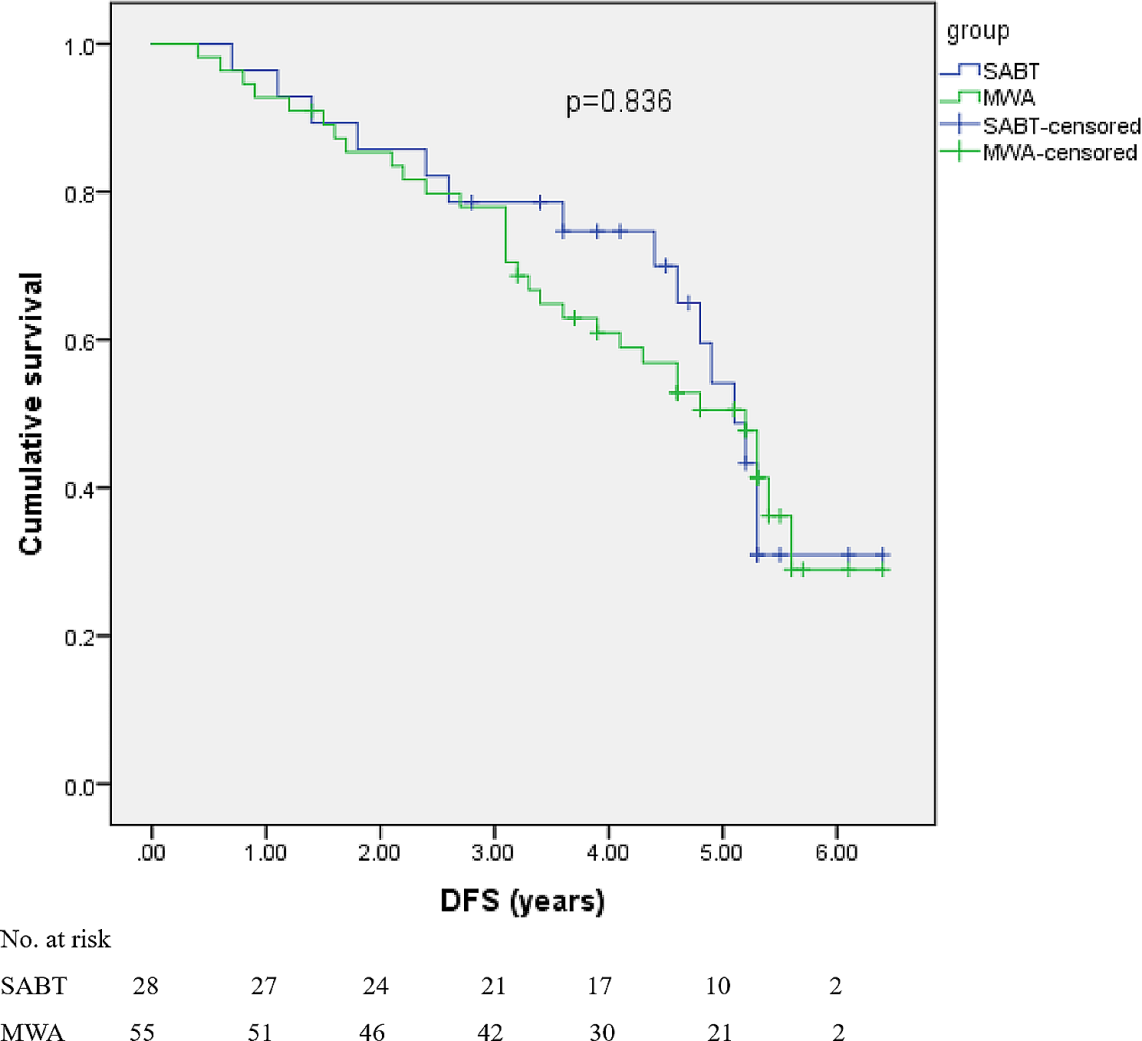
Kaplan-Meier curves of disease-free survival comparison between the SABT and MWA group. There was no significant difference (*p* = 0.836)

The 1-, 3-, and 5-year cumulative OS rates were 96.4%, 82.3%, and 68.4%, respectively, in the SABT group, and 96.4%, 79.7%, and 63.2%, respectively, in the MWA group. The 1-, 3-, and 5-year DFS rates were 92.9%, 74.6%, and 54.1%, respectively, for SABT; 92.7%, 70.5%, and 50.5%, respectively, for MWA. The OS (*p* = 0.631) (Fig. 4) and DFS (*p* = 0.836) (Fig. 5) showed no significant difference between the two groups.

The results of mixed linear model indicated that the histologic type may exert a significant influence on both overall survival (OS) and disease-free survival (DFS) (*P*<0.05). Conversely, the data failed to demonstrate any statistically significant effect of treatment sites. Detailed results were presented in the Table 2.

**Table 2:** Factors impacting the overall survival and disease-free survival*

Characteristics	OS		DFS	
	Estimate (95% CI)	*P* value	Estimate (95% CI)	*P* value
Histologic type Age Smoking history Gender Tumor size Treatment sites	-1.364 (-2.579 to -0.149) -0.070 (-0.057 to 0.003) -0.004 (-0.634 to 0.624) 0.117 (-0.597 to 0.831) 0.164 (-0.390 to 0.717) -0.008 (-0.919 to 0.904)	0.028 0.080 0.988 0.745 0.557 0.987	-1.455 (-2.757 to -0.153) -0.033 (-0.057 to 0.003) -0.076 (-0.600 to 0.753) 0.181 (-0.585 to 0.947) 0.228 (-0.375 to 0.832) -0.112 (-1.079 to 0.855)	0.029 0.048 0.988 0.745 0.557 0.818

*Analyses were conducted using a mixed linear model with histologic type, age, smoking history, gender, and tumor size as the fixed effects and treatment sites as random effects

### Adverse events

No deaths occurred during the procedure or within 30 days after the procedure in the two groups. Pneumothorax was the most common adverse event, encountered in 13 patients in the SABT group, with 4 patients requiring tube drainage, and 18 patients in the MWA group, with 5 patients needing tube insertion. There was no significant difference between the two groups in this regard (*p* = 0.439). In the SABT group, seed shifting occurred in one patient without any symptoms. Radiation-related adverse events such as radiation pneumonia were not observed during follow-up. One case in the SABT group and three cases in the MWA group experienced common pneumonia after the procedure and were successfully treated with antibiotics. A pulmonary abscess occurred in one patient in the MWA group at the ablation site; percutaneous catheter drainage was performed, and this patient recovered well. Other adverse events included subcutaneous emphysema, pleural effusion, hemoptysis, fever, and pain. The complication rates were similar between the two groups except for hemoptysis. The adverse events are shown in Table 3.

**Table 3:** Adverse events

Characteristics	SABT Group (*n* = 28)	MWA Group (*n* = 55)	*P* value
Mild adverse events			
Pneumothorax Hemoptysis Pain Pleural effusion Fever Subcutaneous emphysema Seeds shifting	9 8 6 3 3 2 1	13 2 9 2 10 0 NA	0.439 0.002 0.562 0.330 0.528 0.111 NA
Moderate adverse events			
Pneumothorax Infection	4 3	5 2	0.478 0.330
Severe adverse events			
Pulmonary abscess	0	1	1.000
Disabling adverse events	0	0	NA
Deaths	0	0	NA

Comparison between two groups was determined by Fisher’s exact test; NA, not applicable

## Discussion

To the best of our knowledge, this is the first study to assess the efficacy of SABT compared with MWA in treating early-stage NSCLC. The OS, DFS, and treatment responses were similar in the SABT and MWA groups. There were no significant differences in adverse events between the two groups. No procedure-related deaths occurred.

Lobectomy and radical external beam radiotherapy (EBRT) are recommended as first-line therapy for resectable and inoperable early-stage lung cancer, respectively [27]. Meanwhile, emerging evidence has shown that percutaneous image-guided ablation could be an effective treatment for early-stage NSCLC [28–31]. Since MWA was first reported for the treatment of lung cancer in 2002 [32], an increasing number of studies indicates that MWA is a feasible option for early-stage NSCLC [11, 12, 33, 34]. Similar OS periods and rates were reported between MWA and SABR for inoperable early-stage primary lung cancer [35]. In a propensity-score weighted cohort, no superior rates of OS and DFS were found with surgery compared to MWA [14].

As a special type of radiotherapy, SABT places the radioactive sources directly and precisely into the malignant tumor. Dose variations caused by tumor movement can be minimized or avoided [17]. With the high radiation dose of 110–160 Gy delivered exactly into the malignant tissue by SABT, complete tumor ablation and eradication may be achieved [15]. Meanwhile, the radiation dose declines rapidly as the half-valence layer of implanted seeds in tissue is very short (iodine-125, 1.7 cm). Thus, the radiation damage to normal tissue is minimal [16]. These unique advantages make SABT a prospective therapy for malignant tumors. The definite curative effect of SABT has been demonstrated by numerous clinical studies [36–38]. More recently, several studies suggested SABT may be an alternative choice for early-stage NSCLC ineligible for lobectomy and SABR [21, 39]. Thus, we conducted this study to verify the clinical outcomes of SABT compared to MWA.

In this present study, the 1-, 3-, and 5-year cumulative OS rates were 96.4%, 82.3%, and 68.4%, respectively, for the SABT group. In the study reported by Ji et al., 99 patients with stage T1–3N0M0 NSCLC were treated with SABT. The 1-, 3-, and 5-year OS rates were 96.7%, 70.1%, and 54.4%, respectively [21]. Our results were better than that; this may be because more than 15.3% of the group were stage T2b and T3 patients in their study. Johannes et al. conducted a comprehensive national cancer database study involving 28,834 patients diagnosed with stage I NSCLC, who underwent treatment with either thermal ablation or SABR. Their investigation revealed estimated overall survival rates at 1 year, 3 years, and 5 years: 85.4%, 47.8%, and 24.6% for thermal ablation, and 86.3%, 45.9%, and 26.1% for SABR, respectively [40]. Results from other studies treating early-stage NSCLC patients with MWA demonstrated that the OS rates at 1, 3, and 5 years could reach 89–100%,43–84.7%, and 16–50%, respectively [14, 33–35, 41]. Comparable or even better results were achieved in the current study.

The concept of “tumor ablation,” which was proposed in-depth in 1997 by the Radiology Society of North America, means the direct release of energy to eradicate tumor cells [6]. SABR, SABT, and MWA are similar in nature in the sense that they all apply physical energy to achieve healing [6]. In this present study, the local control rate was 78.6% and 76.4% in the SABT and MWA groups over the follow-up period, respectively. Distant recurrence comprised a large portion of the cases of recurrence. In a study including 99 patients with stage Ia–IIb NSCLC treated with SABT, the 5-year local control rate was 75.7% for the whole group and 86.6% for stage T1 patients [21]. In another study where SABR was given to 912 patients with early-stage NSCLC, the local control rate was 77.9% after a median follow-up duration of 57.2 months [42]. In a decade-long follow-up study utilizing single-fraction SABR for medically inoperable early-stage lung cancer, 229 patients received treatment at a dosage of 30–34 Gy. The 2-year local control rate stood at 92.7%, accompanied by a median overall survival of 44.1 months. Notably, the incidence of grade 3 toxicities was a mere 0.9% [43]. These findings suggested that single-fraction SABR could present an appealing option for managing inoperable early-stage NSCLC. Moreover, when comparing local control rates, SABT, MWA, and SABR demonstrated comparable efficacy.

The Common Terminology Criteria for Adverse Events (CTCAE) system is commonly used in SABR studies. However, adverse events (AEs) were reported according to SIR AE criteria which is a procedure-related AE system in consideration of these two procedures (SABT and MWA) used in the present study. Similar to the non-procedural CTCAE system, the SIR standards also divide AEs into five levels, including mild events, moderate events, severe events, disabling events, and patient death; this corresponds to Grade 1 to Grade 5 of the CTCAE system. Pneumothorax was the most common complication of this present study in the SABT and MWA groups. Mild (Grade 1 in the CTCAE system) pneumothorax accounted for the majority of AEs. Factors influencing the incidence of adverse events related to puncture included operation time, puncture times, and so forth [21]. The incidence of hemoptysis was higher in the SABT than in the MWA groups, probably owing to more puncture needles used in the SABT group. Lung abscess is a major complication of thermal ablation, with a reported incidence of 1.6% [44]. In this present study, pulmonary abscess occurred in one patient (1.8%) with a history of emphysema in the MWA group. The incidence was similar to that reported previously. Radiation-related toxicities such as radiation pneumonia were not observed in this study. This result should be treated with caution on account of the limited number of patients included in the SABT group.

This study has several limitations. First, it was a retrospective study with a limited number of patients, and the patients that received SABT for early-stage lung cancer were especially few. The reliability of the results may be reduced accordingly. Second, as the patients came from four different centers, selection bias may exist. Third, some patients who developed distant recurrence during follow-up had subsequent chemotherapy or targeted therapy, which may have influenced the outcome. It is difficult to eliminate such confounders and biases outside of a prospective randomized trial.

## Conclusions

SABT provides similar efficacy to MWA for the treatment of stage I NSCLC. SABT may be another choice for unresectable early-stage NSCLC. However, future prospective randomized studies are required to verify these results.

## Data Availability

The datasets generated and/or analysed during the current study are not publicly available due to the rules of our hospital but are available from the corresponding author on reasonable request.
